# Cross-Layer MAC/Routing Protocol for Reliability Improvement of the Internet of Things

**DOI:** 10.3390/s22239429

**Published:** 2022-12-02

**Authors:** Jin-Woo Kim, Jaehee Kim, Jaeho Lee

**Affiliations:** 1 Department of Statistics, Duksung Women’s University, Seoul 01369, Republic of Korea; 2 Department of Software, Duksung Women’s University, Seoul 01369, Republic of Korea

**Keywords:** cross-layer protocol, reliability, end-to-end delay, address allocation, IoT, wireless sensor network

## Abstract

The IEEE 802.15.4 standard is recognized as one of the most successful for short-range low-rate wireless communications and is used in Internet of Things (IoT) applications. To improve the performance of wireless networks, interest in protocols that rely on interaction between different layers has increased. Cross-layer design has become an issue in wireless communication systems as it can improve the capacity of wireless networks by optimizing cooperation between multiple layers that constitute network systems. Power efficiency and network scalability must be addressed to spread IoT. In multi-hop networks, many devices share wireless media and are geographically distributed; consequently, efficient medium access control (MAC) and routing protocols are required to mitigate interference and improve reliability. Cross-layer design is a novel network design approach to support flexible layer techniques in IoT. We propose a cross-layer protocol for the MAC layer and routing layer to satisfy the requirements of various networks. The proposed scheme enables scalable and reliable mesh networking using the IEEE 802.15.4 standard and provides robust connectivity and efficient path discovery procedures. It also proposes a novel address-allocation technique to improve address-allocation methods that cannot support large sensor networks. Simulation results indicate that the proposed scheme could improve reliability and reduce end-to-end delay.

## 1. Introduction

The Internet of Things (IoT) is a system wherein many physical objects can be connected to the Internet using a wireless network. In this regard, different network architectures from heterogeneous communication technologies may be adopted depending on the nature of the services to be implemented (e.g., low data rate or high data rate), operational constraints (e.g., availability of external power sources), and coverage (e.g., long-range or short-range links). For example, if end-to-end communication is not possible due to power limitations and/or radio wave failures, mesh network and short-range communication technologies are preferred. In this case, multi-hop communication between devices can be used to pass information from the control center to the edge of the network. In this regard, the IEEE 802.15.4 standard [1] may be applied. Instead, star networks and long-distance communication techniques are preferred if direct and reliable links between the central hub and all nodes deployed in the coverage area are available. This topology can be adopted especially in most low-power wide-area networks (LPWANs) designed to interconnect battery-driven devices over long distances at low data rates.

These technologies can be used in a wide variety of applications related to buildings, industrial automation systems, health care, smart grids, and security [1,2]. A key concern of IoT is whether Internet protocol technology can be used in limited devices such as sensors and actuators. Devices used in IoT are devices with limited resources in memory, battery, bandwidth, and connectivity [3]. The Internet Engineering Task Force (IETF) formed a working group (WG) in 2013 and began standardizing activities to support the requirements of the IoT. As the IoT relates to various technologies, the IETF has working groups focused on four IoT areas—that is, the Internet, applications, routing, and security. Typically, there are low power wireless personal area network (6LoWPAN) WGs that have ended their current activities in the Internet area, constrained restful environments (CoRE) WG in the application area, routing over low-power and lossy networks (ROLL) WG in the routing area, and authentication and authorization for constrained environments (ACE) WG in the security area. The IETF WGs are actively working on standardizing IoT-based protocols to be used within low-power and lossy networks (LLNs) comprising wireless nodes with constraints in power and computing capacity.

Another key feature that IETF pays attention to in IoT standardization activities is allowing a wide range of “things” to use interoperable technologies to communicate with each other. Here, “things” range from built-in sensors to complex machines (e.g., cars) or even large infrastructure (e.g., bridges). Currently, deployable IoT stacks can easily configure many additional protocols, including protocols not designed for IoT or IETF (e.g., ISO Standard Message Queuing Remote Transport (MQTT) or IETF Standard HTTP). This is mainly due to the fact that IoT is important and suitable for various numbers of definitions, design visions, and deployments [4].

Recently, as research on smart cities, a national strategic project, has begun in earnest, the scope of application of IoT technology is expanding even more. One of the goals of the smart city concept is to monitor, control, and manage resources such as electricity. In this regard, the starting point for many current smart city implementations in Europe is the public lighting system [5,6]. In [7], the authors focus on the design of streetlight controllers to reduce power consumption and ultimately reduce public lighting costs.

There are studies that have adopted IEEE 802.15.4 technology in smart city scenarios. In [8], the authors discuss simulators for evaluating the performance of IEEE 802.15.4 and IEEE 802.11 when applied to such scenarios. In [9], the authors investigate the deployment of wireless sensor networks at road intersections in some of the world’s major cities. The authors show that the resulting graph is highly disconnected and comprises up to 25% isolated nodes, using propagation models corresponding to 2.4 GHz IEEE 802.15.4 technology and considering 52 city maps extracted from OpenStreetMap. One of the largest test beds related to smart cities is the Smart Santander [10], with approximately 3000 IEEE 802.15.4 devices deployed.

The effects associated with these applications are not limited to economic aspects, but are also the ability to construct and use capillary wireless networks that reach all locations with smart streetlights. Through this, the following new services can be provided.

⚬ Smart Parking: Real-time monitoring of parking lots (parking/user) [11,12,13,14,15,16,17,18]
⚬ Waste Management: Detecting waste levels in containers to optimize waste collection paths and schedules [19,20,21]
⚬ Air Quality: Air Pollution Monitoring [22,23,24,25]
⚬ Structural integrity: monitoring vibration and material conditions of buildings, bridges, and historical monuments [26,27,28]
⚬ Traffic monitoring and control: traffic jam detection and alternate path notification [29,30,31,32,33,34].

Recently, an Zigbee S2C module that expands the communication distance of Zigbee has been released. The Zigbee S2C has a range of 1.2 km and the Zigbee Pro S2C model is 3.2 km. The data transmission rate is 250 kbit/s for RF and the serial portion is 1 Mbit/s. There are 16 communication channels of Zigbee S2C and 15 communication channels of Zigbee Pro S2C [35]. The Zigbee S2C module is a very low-cost communication module suitable for streetlights installed at tens of meters apart.

Although existing Internet-related technologies on wireless network protocol can be applied to the IoT, many limitations have been expressed in technical applications as related applications have become remarkably diverse. In particular, various medium access control (MAC) and routing technologies have been proposed considering the IoT requirements. For example, IoT applications with low-power requirements use the IEEE 802.15.4 standard or associated extensions of it as the physical or media access control layer [36]. The IEEE 802.15.4 standard, a wireless personal area network (WPAN) standard, uses frequencies in the 2.4 GHz unlicensed band and has the advantage of being able to communicate at low power and low cost. Applications—such as building and industrial automation systems—use network technologies based on the IEEE 802.15.4 standard, such as ZigBee and WirelessHART. Recent market research shows that these standards account for approximately 50% of the building and industrial automation systems market [1]. By contrast, routing protocol technology still has no dominant solution. To address this problem, the IETF is working on standardizing routing protocols based on IEEE 802.15.4 MAC.

A crucial challenge for wireless MAC and routing protocols is to simultaneously satisfy the diverse network requirements of the IoT, interlayer interoperability, and standardization requirements [37]. In particular, the complex interactions between these MAC and routing layers must be considered in depth for critical IoT applications that have high-performance requirements, as traditional layer-separated research and development inevitably has many performance limitations [38]. For example, when the IoT is applied to a control system, the information at the source node must be delivered smoothly to the target node to ensure the stability of the overall control system. Consequently, performance measures such as the reliability, delay, and power consumption of the wireless network should be considered in depth. A fundamental consideration of the interaction between network layers is essential to satisfy these performance requirements.

**Figure 1:**
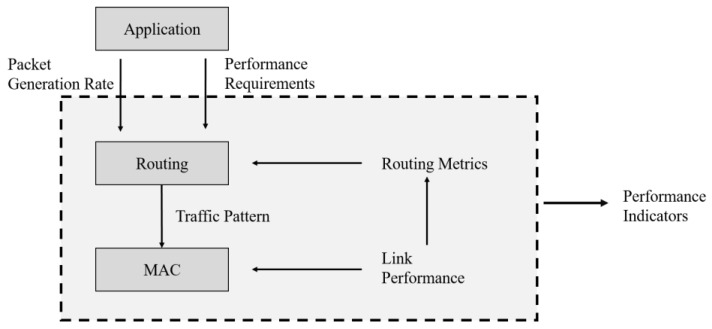
Modeling and design based on MAC and routing layer interaction.

Figure 1 shows the relationships between the MAC and routing layers using a block diagram. Information such as the topology of the application layer and the volume of traffic generated by the node becomes the input data to the entire system.

The network routing manager considers these system inputs and routing performance metrics to distribute traffic to the nodes in the network and forward data packets. Typical routing performance metrics include the end-to-end reliability, transmission latency, and overall network energy consumption. These routing performance metrics can be directly affected by the MAC technology because different MAC protocols exhibit different reliability, delay, energy consumption, and traffic congestion metrics for each link. Consequently, when MAC and routing layer technologies are designed independently in the feedback loop (Figure 1), it can be difficult to simultaneously satisfy various requirements such as reliability and delays.

High reliability and low latency transmission are very important factors in wireless sensor networks, and many studies are being conducted [39]. In a typical routing protocol that ensures reliability, a routing table with a calculated hop count is configured so that the shortest hop can be statistically selected. However, link failures, node mobility, and routing path changes make reliable data transmission difficult.

In this paper, we proposed a solution to support the reliability, scalability, and robustness of multi-hop IoT environments. We designed a MAC layer-based cross-layer protocol termed Topological Structure Mesh Routing (TSMR) protocol The proposed scheme provides robust connectivity and efficient route discovery procedures from the gateway to mobile and static leaves, and vice versa. Additionally, the proposed scheme provides fast and efficient association/reassociation procedures for the mobile nodes while limiting the impact of the signaling overhead to support the device’s mobility.

## 2. Background

### 2.1. Overview of the IEEE 802.15.4 Standard

**Figure 2:**
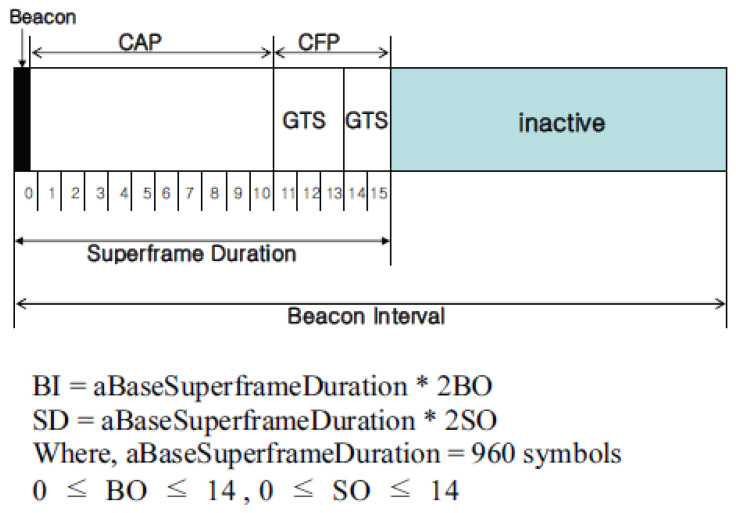
The superframe structure of the IEEE 802.15.4 standard.

The superframe structure of the IEEE 802.15.4 standard is shown in Figure 2. Superframes are divided into active and inactive sections. In addition, the active section consists of 16 slots, of which the beacon, which serves to synchronize nodes, is transmitted in the first time slot. It has a structure that can adjust the active and inactive parts by adjusting the beacon interval (BI) and the superframe duration (SD).

IEEE 802.15.4 MAC defines two access control methods—that is, competition-based unslotted CSMA/CA methods and hybrid-based beacon-enabled modes, which include slotted CSMA/CA and guaranteed time slot (GTS) allocation methods. When attempting to transmit a packet using the slotted CSMA/CA method, each node initializes four parameters—that is, the number of backoffs (NB) in the MAC layer, competing window length (CW), backoff exposure (BE), and retransmission number (RT).

The MAC layer generates a random number between units [0, 2^BE^–1], and waits until the generated backoff time ends. When the backoff time is over, the node monitors the channel state through clear channel assessment (CCA). If the CCA is idle, the node starts sending packets, and if the channel state becomes busy and the CCA fails, the MAC increases the NB and BE by one to the maximum values of *macMaxCSMABackoffs* and *macMaxBE*. If the BE value reaches *macMaxBE*, the maximum value is maintained until reset. If the NB value is greater than *macMaxCSMABackoffs*, the packet is discarded as a channel access failure. If not, the CSMA/CA algorithm uses a random number related to the backoff and repeats the process. If a node’s channel access is successful, it waits to transmit a packet and receives an ACK message. When the ACK corresponding to the transmitted packet is received, it can be considered that the packet has been successfully transmitted. If a packet is not received during a packet collision or ACK timeout, the RT variable increases one by one to the maximum value of *macMaxFrameRetries*. If the RT value is less than *macMaxFrameRetries*, the MAC initializes BE = *macMinBE* and repeats the CSMA/CA for channel reaccess.

IEEE 802.15.4 can use either 16-bit or 64-bit MAC addresses, mainly using a 16-bit short address system with low overhead, for identifying nodes within a wireless sensor network. The 64-bit extended unique identifier (EUI) provides a unique address system for sensor nodes. The advantage of the 16-bit short address is that it has less overhead when compressing the header. However, because it is not an address uniquely assigned to the sensor node, address duplication may occur, and the address may change due to link changes or network movement of the node. That is, network management is difficult by making individual discrimination and access of sensor nodes impossible in a wireless sensor network. The 64-bit EUI address allocation has a unique ID, and the address of the sensor node is maintained even when the link changes and moves, but the 64-bit address is used for header compression even in simple communication between sensors, so the overhead is larger than that of short address. In addition, EUI-based address generation does not provide a hierarchical concept address that can be used for hierarchical tree routing, which can be performed with low power–low memory resources. Therefore, it is necessary for the reliable and stable operation of wireless sensor networks to save limited resources of wireless sensor networks by reducing header size while maintaining existing addresses even when changing or moving links.

### 2.2. Related Work

The 6LoWPAN standard [40,41] proposes a routing technique with a hierarchical structure for 6LoWPAN (HiLow) [42], and the ZigBee [43] standard outlines the ZigBee tree routing (ZTR) structure. As network protocols with hierarchical structures allocate addresses with structural patterns to devices, complex calculations or processes are not required to establish the routing paths. Consequently, they are advantageous for networks consisting of devices with constrained resources.

The collection tree protocol (CTP) [44] is a routing protocol that considers unstable link quality and dynamically changing topologies in ad-hoc networks with low data rates. However, the CTP has the disadvantage of adding a network load by broadcasting periodic hello messages to check link quality and routing paths. Additionally, because it is a tree-based routing technique, data transfer from sensor nodes to sink nodes is possible, whereas other additional tasks are required for routing between neighboring nodes.

The link ordering-based data gathering protocol (LINKORD) [45], which works similarly to the CTP, proposed a new path cost function to prevent path construction that required more than the maximum number of transmissions allowed by the link layer. The proposed path cost function could construct a routing path with high throughput considering the data transmission probability and relative location of the link.

The general self-organized tree-based energy-balance routing protocol (GSTEB) [46] extends the life of the wireless sensor network (WSN) by balancing energy consumption. Tree-based routing techniques can reduce the resources required to establish and maintain the routing routes. However, for P2P traffic to communicate with adjacent nodes, tree-based routing techniques bypass packets through nearby nodes, which can create a bottleneck at the root peripheral node and reduce the packet transmission rate.

Efficient routing protocols for P2P traffic have also been proposed. The 6LoWPAN ad hoc-demand distance vector routing (LOAD) protocol [47] simplifies and optimizes the ad-hoc on-demand distance vector (AODV) routing protocol to suit the 6LoWPAN environment. Because the LOAD protocol performs routing path discovery immediately before data transmission, the algorithm can provide an almost optimal path for sending P2P traffic to neighboring nodes. The AODVjr protocol is a simplified version of the AODV protocol that is widely used in the Zigbee network routing algorithm, which reduces energy consumption and is easy to realize [48]. However, these algorithms are not suitable for resource-constrained devices because they generate many control packets for flood-based path discovery and consume considerable resources to maintain routing tables.

Shortcut tree routing (STR) algorithms [49,50,51] and enhanced hierarchical routing protocols (EHRPs) [52] are tree-based routing protocols that can efficiently transport P2P traffic. Instead of forwarding packets along the tree path, they can reduce hop distances by forwarding packets to nodes with the smallest remaining tree distance among the neighboring nodes. Enhanced STR (ISTR) [53] improves the P2P performance of STR by reducing hop counts and alleviating congestion using enhanced beacon frames and extended neighboring node tables. However, because they use tree topologies, when the tree structure is broken owing to link failure, they incorrectly predict the tree distance. Similarly, these tree-based routing protocols can affect the entire network if they fail along a path near the root.

The IPv6 routing protocol for low-power and lossy networks (RPL) is an IPv6 routing protocol under standardization in the ROLL WG of the IETF. RPL is designed for low-power and noisy network environments such as IEEE 802.15.4, power line communication, and supports a variety of routing metrics to accommodate the diverse requirements of several applications. To this end, functions corresponding to routing metrics and path optimization to achieve the requirements of each application were separated as objective function (OF), and the contents defined in the standard define general contents commonly used for various OFs [54]. There are many routing protocols that enhance RPL. In [55], the authors proposed a modification protocol to enable cluster trees in IEEE 802.15.4 to work efficiently with RPL. Based on this modified MAC layer, the authors used an opportunistic forwarding scheme [56] to extend the RPL capable of forwarding packets. Instead of always using the preferred parent, the node opportunistically forwards the packet through another parent as long as the sink path is better. In [57], the authors propose a proactive multipath load-balancing approach that seeks to achieve maximum life by equalizing traffic rates between nodes at the same distance in the local sink. In this algorithm, load balancing is performed through routing graphs generated by an Multi-class Multipath Routing Protocol (M2RPL) for low-power and lost networks. In [58], the authors proposed a multipath RPL (MRPL) algorithm to maximize network life and minimize total transmission costs. The algorithm proposed a novel heuristic load balancing mechanism (HeLD) to find the optimal traffic rate for each link and simultaneously equalize the rate of energy consumption between nodes of the same depth. In [59], the authors proposed a multipath routing protocol to provide efficient event packet forwarding in event-based wireless sensor networks using different mechanisms in the path-building and data-transfer phases. However, these multipath routing protocols only consider traffic flows to the sink, such as MP2P traffic. Accordingly, a bypass problem occurs during P2P communication, and packets converge around the sink.

Mesh-topology-based routing protocols offer promising techniques to overcome these reliability problems. Fault tolerance for a single-path failure can be achieved by maintaining both basic and alternative paths.

Wireless mesh networks have been considered promising access networks for next generation technology, providing better services and guaranteeing flexibility and robustness to different wireless network environments. The mesh network can take several paths from the source to the destination using the intermediate nodes within the network.

Riggio et al. [60] proposed a hybrid architecture that combined a WSN and a wireless mesh network (WMN). In [40], the sensor node only used resources (i.e., power) to implement sensing functions, whereas the mesh router collected encrypted data and then relayed the aggregated data to the sink, reducing the volume of traffic exchanged over the network and the resulting overall power consumption.

Muthukumaran et al. [61] proposed a distributed beacon scheduling-based MAC protocol for the IEEE 802.15.4 beacon mode. The proposed algorithm enabled low-power mesh networking through distributed beacon scheduling and was compatible with the IEEE 802.15.4 standard. Furthermore, the proposed scheme was able to improve the reliability of the IEEE 802.15.4 cluster-tree topology (which provided only a single communication path) by constructing a mesh topology.

The ISA 100 standard was designed to provide reliable and safe behavior in the field of system monitoring and control [62]. It defines the structure of networks, systems management, gateways, security managers, etc., for low-speed wireless connections to fixed, portable, and mobile devices that support limited power consumption conditions. The ISA 100 standard is fully compatible with the IEEE 802.15.4 standard, but it achieves similar results through simpler messages that use the data link layer subnet time shared as a security element without IEEE security.

Francesco et al. [63] proposed an adaptive access parameter tuning (ADAPT) algorithm based on analytical studies of the IEEE 802.15.4 standard. ADAPT is suitable for resource-constrained sensor nodes because of its low complexity, as it uses only local information on sensor nodes for network configuration and operation.

The IEEE 802.15.5 standard provides mesh capabilities for both high- and low-rate wireless personal area networks [64]. Low-rate meshes can be built on IEEE 802.15.4 MAC, with high-speed meshes being built on IEEE 802.15.3 MAC. The IEEE 802.15.5 standard provides basic mesh capabilities, including tree formation, address block allocation, and mesh routing. For low-rate sections, several important advanced features, including multicasting, power saving, and portability, have been presented. High-rate sections provide server-guided routing to support QoS provision. However, this standard involves changing the MAC superframe structure, which affects interoperability with the current MAC standard.

Silva et al. [65] proposed a methodology based on the automatic generation of fault trees to evaluate the reliability and availability of WSNs when permanent faults occurred in network devices. The proposed methodology supported all topologies, varying levels of redundancy, network reconfigurations, device importance, and arbitrary failure conditions. The proposed methodology was particularly suitable for the design and verification of WSNs when attempting to optimize the reliability and availability requirements.

In [66], the authors proposed a novel multi-path routing protocol method for event-driven WSNs that provided reliability, load balancing, and energy efficiency for routing paths. Furthermore, using residual energy as the main parameter for dynamic switching between two alternative paths, the algorithm could be deployed for the virtualization of WSNs.

Wei et al. [67] proposed a low-power grid-based cluster-routing algorithm for WSNs. A feature of the algorithm was that it configured nodes in the grid in a clustering manner. The clustering head was dynamically selected considering the energy consumption of the cluster node, before communicating with the base station through the relay node.

Shabani et al. [68] proposed a smart Zigbee/IEEE 802.15.4 MAC protocol for wireless sensor multi-hop mesh networks based on a beacon-enabled mode. The proposed algorithm could build a smart Zigbee/IEEE 802.15.4 MAC-based beacon-enabled mesh network while improving the performance of the network in terms of time delay, duty cycle, energy saving, and reliability.

Compared with existing studies, our proposed algorithm has the following characteristics: (1) The TSMR deals with the mobility of multi-hop WSNs based on the IEEE 802.15.4 standard, a still unresolved problem to the best of our knowledge. (2) The TSMR can find the optimal path with reduced computational overhead. (3) The TSMR provides an efficient and distributed address-allocation algorithm. (4) The TSMR improves the network reliability.

## 3. Topological Structure Mesh Routing

Energy consumption is one of the major problems in wireless communication systems, including the IoT. To address this, the IEEE 802.15.4 standard improves energy efficiency through time synchronization using a beacon frame broadcast by a coordinator. The IEEE 802.15.4 standard defines two types of devices—that is, a full function device (FFD) and a reduced-function device (RFD). FFDs can operate in three modes—namely, the coordinator, router, and device modes. Additionally, FFDs can be network coordinators without restrictions on the network topology configuration and can communicate with all the equipment on the network. Conversely, RFDs operate only in device mode and can only participate in a limited form of star topology. Additionally, RFDs cannot act as network coordinators and can only communicate with the parent router.

The IEEE 802.15.4 standard defines only one-hop communication among adjacent devices. Therefore, to support multi-hop communication, it provides a cluster-tree topology in which most devices are FFDs. RFDs may be associated with a cluster-tree network as leaf nodes because they can only communicate with the parent router, or FFD. The cluster-tree topology is capable of low-power consumption while performing multi-hop communication. However, in a cluster-tree topology, the entire network may be shut down because of the departure of a few router devices. The IEEE 802.15.5 standard address-allocation method can assign a unique address to all nodes throughout the network, but the disadvantage is that it takes some time to form an initial network in a large network and does not properly respond to the dynamic addition and deletion of nodes. Moreover, the memory overhead for storing the addresses of all the nodes increases.

The ZigBee protocol, established by the ZigBee Alliance—a leading standardization organization on WSNs—provides a distributed address-allocation technique that sets up a balanced tree-based address area and then configures a tree topology to allocate addresses. Currently, the distributed address allocation scheme using the Zigbee standard Cskip starts with a maximum tree depth (Lm), a maximum number of children (Cm), and a maximum number of routers (Rm) specified in advance. This has the advantage of simplifying routing by using only address information without requiring a separate routing table or route search process while ensuring the uniqueness of the address when a new node enters the network. However, the ZigBee address allocation scheme cannot support asymmetric networks. This is because all devices in the tree network use the same network parameters of Cm, Rm, and Lm values, and therefore use the same Cskip. Moreover, the value of the maximum network depth (Lm) is often very difficult to select before actually forming a network. When applied to a large-scale network, the predetermined parameters of Cm and Rm cannot always meet the address allocation requirements, which results in some nodes not being able to obtain network addresses and becoming orphaned [69,70,71,72]. Additionally, owing to the use of a fixed topology, an orphan problem can occur that cannot be accessed because of the exhaustion of addresses of neighboring routers, even though sufficient address areas remain in the network. An address-allocation scheme for large-scale networks should be able to shorten the initial network formation time, avoid generating excessive traffic in the network, and effectively respond to the dynamic addition and removal of nodes.

If the destination node locates the subtree of an intermediate node, the routing protocol of the cluster-tree network should find the next-hop node for a given destination address without a routing table. However, the sender node does not know whether the destination is located nearby or is not within the subtree itself. Consequently, cluster-tree-based routing cannot find multi-path routes between the source and destination devices and cannot optimize the routing path.

**Figure 3:**
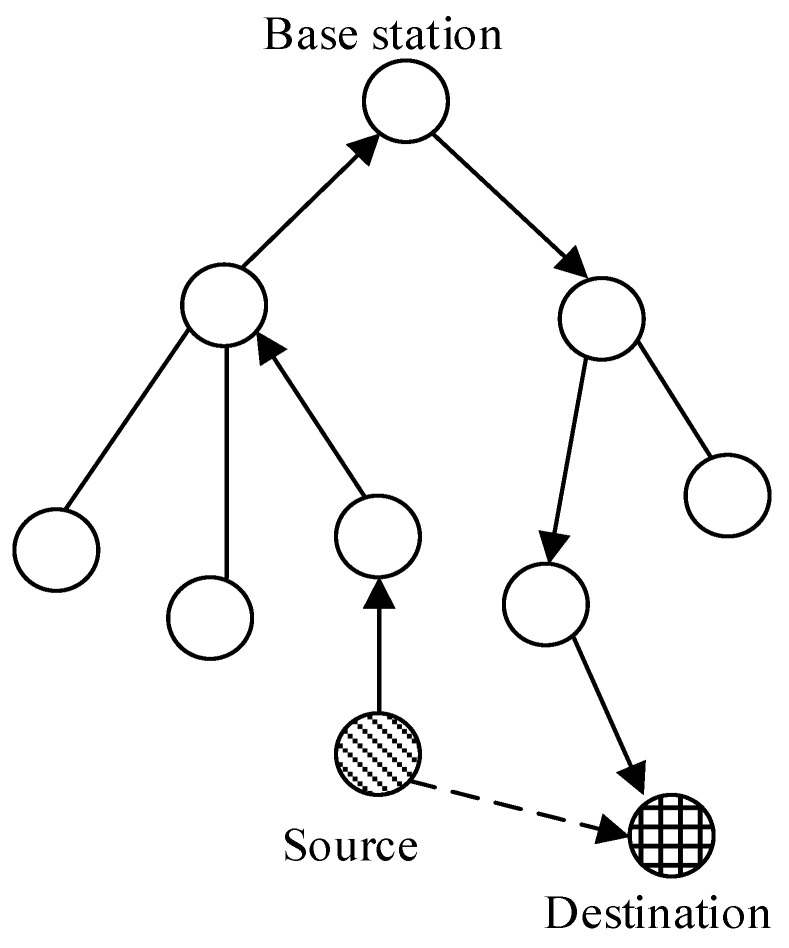
Drawbacks of the cluster-tree protocol.

Figure 3 shows the drawbacks of the cluster-tree-based routing protocol.

In Figure 3, the source transmits a data frame through the base station, although the destination is in the communication range of the source device. Additionally, when the mobile device moves to another cluster, it transmits the reassociation frame to the base station and allocates a new address from the base station. To address these drawbacks, we proposed a new MAC layer-based cross-layer protocol for mesh routing.

### 3.1. Address Assignment

This section describes the proposed address allocation mechanism. It is to find the best path to send the most important data frames in the network layer to the destination in a multi-hop network. In this paper, we propose efficient routing using (x, y, z) coordinate values. The TSMR can improve the energy efficiency used in each node by reducing the waste of address space compared to the standard method.

The network frame format established by the ZigBee Alliance uses a total of 16 bits of address space for FFD devices and can allocate a total of 65,536 nodes. However, in a wireless communication environment, the components for space must be fully considered, and if 65,536 allocated nodes are not met, it results in a waste of address space. To solve this problem, a 16-bit address block range can be allocated as much as necessary under the administrator’s authority, such as (8, 4, 4) or (4, 4, 4), to each of the (x, y, z) coordinate values. In the case of routing in a wireless communication environment, routing costs are required according to how many hops it passes. Therefore, it can be said that the smaller the number of hops within the limit of communication, the more efficient the routing technique.

**Figure 4:**
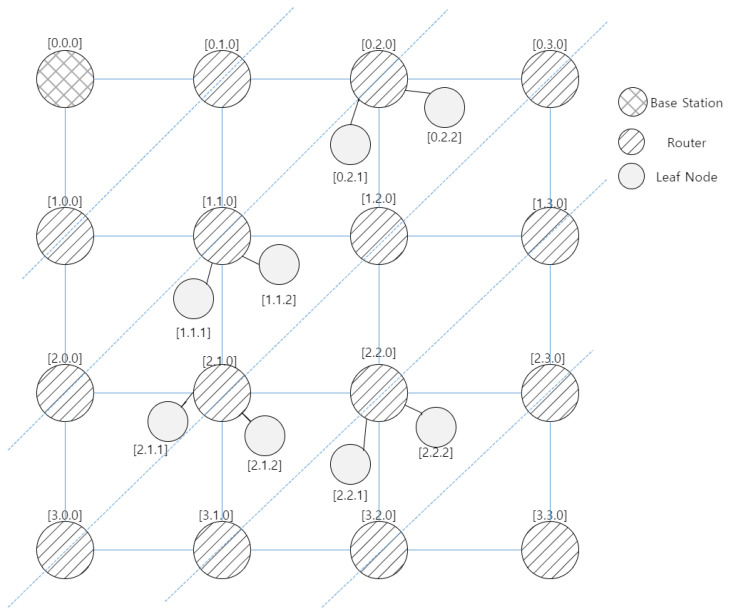
Proposed network address-allocation scheme.

Figure 4 illustrates the proposed address-allocation scheme. In Figure 4, the address of each device in the TSMR consists of a row ID, column ID, and leaf ID. The row and column IDs indicate the hop counts in the row and column directions from the base station [0.0.0], respectively. For example, the device with address [1.1.0] is the router located at a distance 1 hop in the row and column directions from the base station. In other words, the proposed address refers to the relative location of the base station. In the TSMR, the leaf IDs of both the base station and router are set to 0, and the leaf ID of the leaf node is arbitrarily allocated by each router. Using the proposed addressing scheme, the source device can transmit a data frame to its destination without the help of a routing table. The detailed address-allocation scheme is as follows:

- For the first time, the base station that receives the association-request frames from the FFDs selects two devices with the best RSSI and allocates two addresses—that is, [1.0.0] and [0.1.0]. The two routers that receive the association-response frame, [1.0.0] and [0.1.0], from the base station join the base station PAN.
- The device that receives the beacon frame from the two routers with addresses [1.0.0] and [0.1.0] transmits the association-request frame—including the RSSI value of each received beacon frame—to the two routers. The device receives the beacon frame from only one router—that is, the association-request frame—including the RSSI value of the received beacon frame to the router. The two routers [1.0.0] and [0.1.0] transmit the association-relay frame, including the information of the received association-request frames, to the base station. The base station that receives the association-relay frames from the two routers allocates a new address [1.1.0] to the device with the best RSSI value. Additionally, it selects the device with the best RSSI among the devices that transmit the association-request frame to the router [1.0.0] and allocates a new address [2.0.0] to the selected router. In the same manner, the base station allocates a new address [0.2.0] to the router.
- Similarly, the base station selects the device with the best RSSI among devices that transmit the association-request frames to two routers [x.y.0] and [(x + 1).(y − 1).0] and allocates a new address [(x + 1).y.0] for the selected devices.

As mentioned above, each router allocates a leaf ID with a non-zero value to the leaf nodes, the leaf ID being determined by the order in which the router adds them. In the TSMR, the address to which the leaf node is assigned is determined by the address of the parent router, and the base station is not involved. Accordingly, the TSMR may reduce the burden of the base station due to address allocation and management.

### 3.2. Initial PAN Construction

In this study, we assumed that the base station was connected to a personal computer via a USB/serial link to connect with the external network and become the first personal area network (PAN) coordinator in the mesh network. The coordinator that joined the mesh network is referred to as the router. In the TSMR, the FFD can operate as a router or leaf node, whereas the RFD can only operate as a leaf node and is connected to the router.

**Figure 5:**
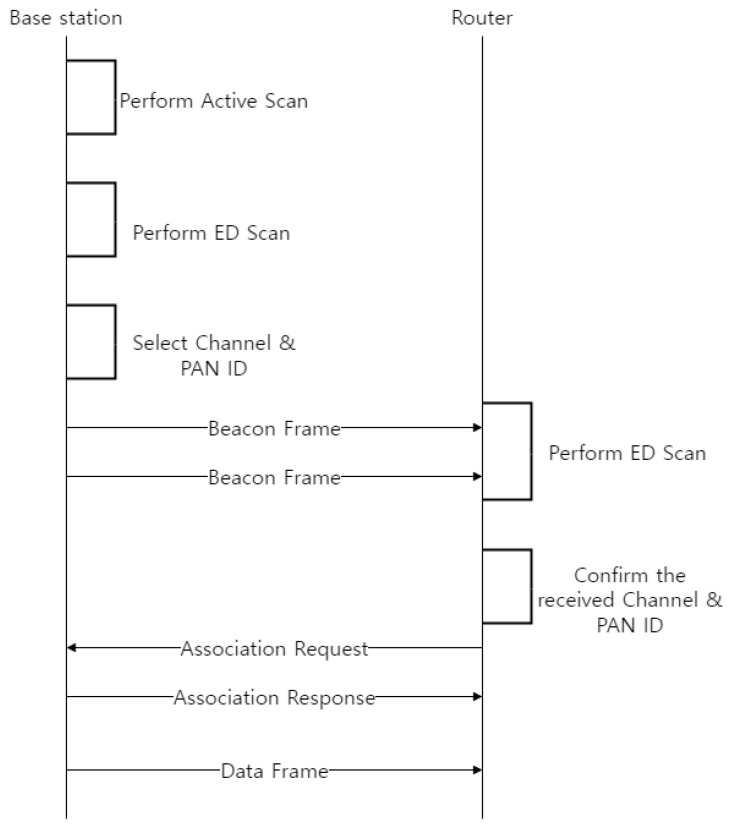
The timing diagram for constructing the PAN in the TSMR.

Figure 5 shows the timing diagram to configure the mesh network by the base station and router in the TSMR. As shown in Figure 4, the base station collects the network information including PAN ID and channel, and it selects non-overlapped channel and ID as PAN ID and channel. Then, the base station broadcasts the beacon frame which includes the information of the selected channel and PAN ID to the adjacent PAN router. The router that receives the information of the selected channel and PAN ID performs passive scan. The router transmits the association request frame. When the router receives the association response frame from the base station, it configures the mesh network and communicates with neighbor devices.

The base station collects the network information—including the PAN ID and channel—and selects the non-overlapped channel and ID as the PAN ID and channel, respectively. It then broadcasts the beacon frame—which includes the information of the selected channel and the PAN ID—to adjacent routers. The router that receives the channel information and PAN ID selected by the base station performs a passive scan. If the channel selected by the base station does not overlap with the channel used by the adjacent PAN or device, the router transmits an association-request frame, the base station receiving the association-request frame during the predefined beacon interval (BI). When the base station receives the association-request frame, it determines the received signal strength indicator (RSSI) field in the association-request frame and compares the values of the RSSI fields received from other routers. The base station then allocates the superframe durations (SD) to the routers that transmit the association-request frame. In this case, it allocates the closer SD of the base station to the router with the higher RSSI value. Subsequently, the base station transmits the association response frame—which includes the information of the allocated SD—to the routers. When the router receives an association-response frame from the base station, it completes the initial PAN construction process.

**Figure 6:**
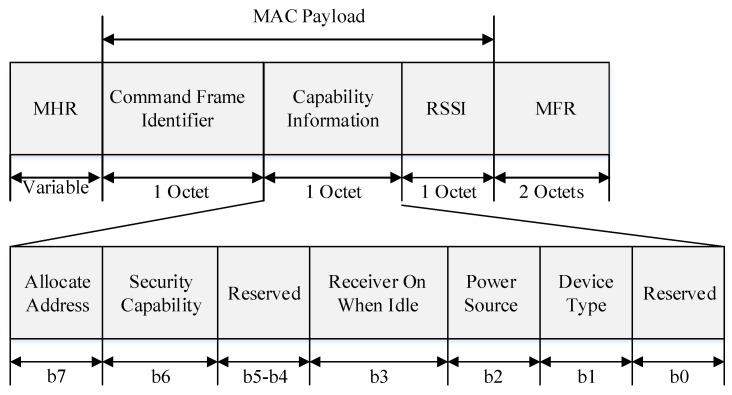
Format of the proposed association-request frame.

Figure 6 shows the proposed association-request frame format.

In Figure 6, the *device-type* field is used to distinguish between the FFD and RFD. If the device is an FFD, it sets the *device-type* field to 1. If not, the *device-type* field is set to 0 to indicate an RFD. When the device receives power, it sets the *power-source* field to 1. If not, the *power-source* field is set to 0. If the device does not turn off the receiver to save power during the idle period, it sets the receiver to an idle state when the *idle* field is 1. If not, it sets the receiver to an active state when the *idle* field is 0. If the device can send and receive encrypted MAC frames, the *security-capability* field is set to 1. If not, it sets the *security-capability* field to 0. If the device wants to be assigned a short address after joining the PAN, it sets the *allocate-address* field to 1. If not, the *allocate-address* field is set to 0. The RSSI field indicates the RSSI value of the frame received from the base station or router.

**Figure 7:**
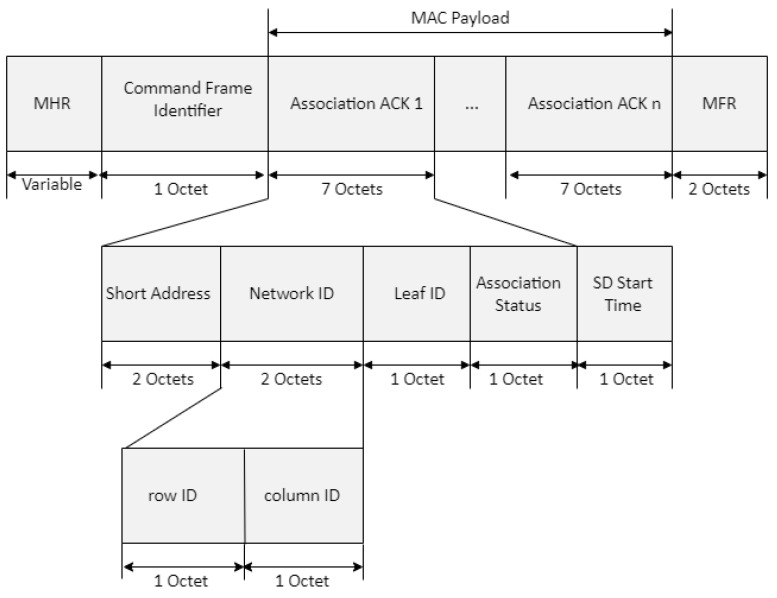
Format of the proposed association-response frame.

Figure 7 shows the proposed association response frame format. In Figure 7, the *network ID* field indicates the MAC address of the router and consists of the row and column ID. The *short-address* field indicates the MAC address of the leaf device. The *leaf-ID* field refers to an ID assigned by the leaf node from the router, the *leaf-ID* fields of the base station and router being set to 0. If the base station is not able to associate the device with its PAN, the *short-address* field is set to 0xff, and the *association status* field contains the reason for association failure. If the base station or router can associate the device with its PAN, this field contains the short address that the device may use in its communications on the PAN until it is disassociated. A *short-address* field value equal to 0xfe indicates that the device has been successfully associated with a PAN but has not been allocated a short address. In this case, the device communicates with the PAN by using only its extended address.

### 3.3. Network Expansion into a Mesh Topology

**Figure 8:**
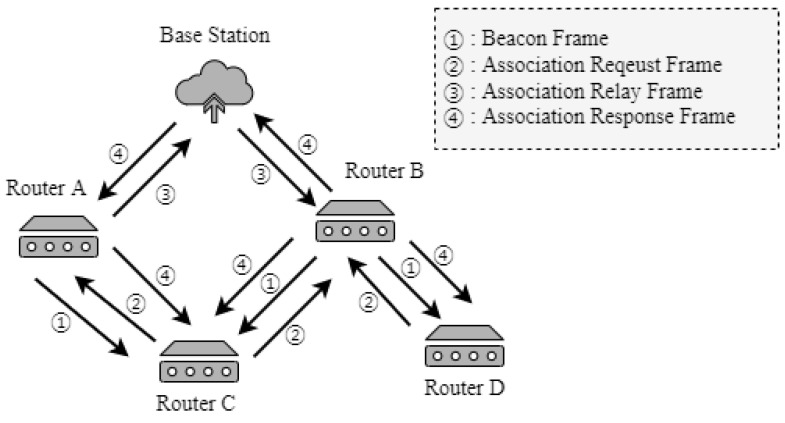
Process of the proposed network expansion.

Figure 8 shows the process of network expansion into a mesh topology using the TSMR.

In Figure 8, routers A and B are associated with the PAN constructed by the base station using the TSMR. After joining the PAN, the routers broadcast beacon messages periodically. Routers C and D, which are not yet associated with the network, receive beacon messages from routers A and B. On the receipt of the beacon message, router D transmits the association-request frame to router B, and router C transmits the association-request message to routers A and B. Router A transmits an association relay message that includes the information of the association-request message received from router C. Router B, which receives association-request frames from two or more routers, aggregates the association-request frames and transmits the association-relay frame to the base station. If the base station receives the association-relay frame, it selects the SD for the router during the inactive period and transmits the association-response frame. The base station selects the SD for the router based on the RSSI field included in the association relay frame or request frame. If the router receives the association response frame, it determines the *short-address* field in the association-response frame. If the *short address* field of the received association response frame is the same as its own address, the PAN router initiates communication on the assigned SD.

**Figure 9:**
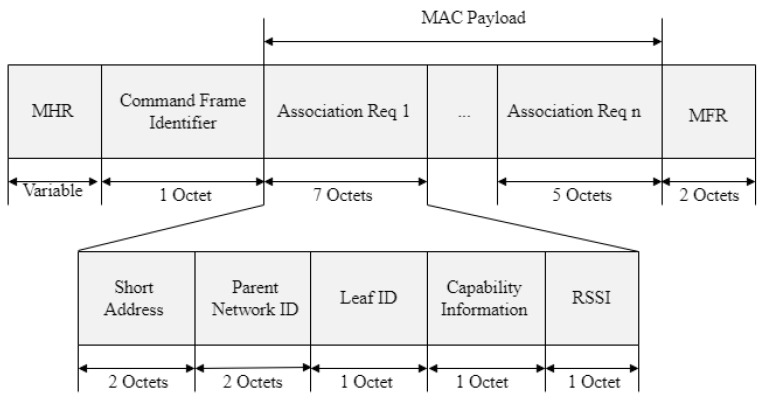
Format of the proposed association-relay frame.

Figure 9 shows the format of the proposed association-relay frame.

In Figure 9, the *parent-network ID* field represents the network ID of the parent router that received the association-request frame and consists of the raw ID and the column ID. The *short-address* field indicates the MAC address of the device that transmits the association-request frame. The *leaf ID* field points to the address assigned to the leaf device. The *RSSI* field is set to the RSSI value measured when the router receives an association-request frame.

**Figure 10:**
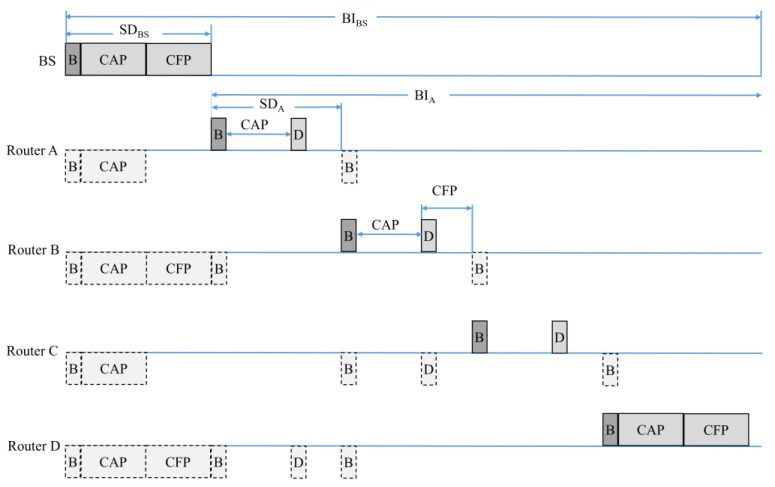
Data flow of the mesh network configured using the TSMR.

Figure 10 shows the data transactions of mesh networks configured using the TSMR. In Figure 10, base station BS broadcasts a beacon frame at the superframe starting point. The neighboring router synchronizes with the base station using a beacon frame received from the base station. The router then broadcasts beacon frames in the beacon intervals. All routers receive beacon frames from neighboring routers and determine whether data frames should be received from neighboring routers. If there is no data frame to receive from the neighboring router, the base station and the router switch to sleep mode during the corresponding SD to reduce energy consumption. In addition, to avoid data collisions and congestion, all routers transmit data frames to neighboring routers in the contention free period (CFP). In Figure 10, when Router A transmits data to Router D, Router A uses a beacon frame to notify Router D of the data transmission. Router D may receive the data frame from the CFP allocated by Router A. All routers except A and D switch to power-saving mode during that interval to reduce energy consumption. Router B broadcasts a beacon frame including CFP allocation information when transmitting a data frame to Router C. Router C receives the data frame from Router B during the allocated CFP using the Beacon frame received from Router B.

### 3.4. Routing

The TSMR is based on a simple routing strategy in which the next route in the path is selected based on a hop count using the new addressing scheme. The source device calculates the distance from it to the destination using the row and column ID. After calculating the distance, it selects the neighboring router with the closest distance between the source and destination devices and transmits the data frame to the selected router. The same process is repeated until the data frame is delivered to the destination device. The detailed routing method is as follows:

a. The source device or routers that receive the data frame subtract their own address [R1.C1.0] from the address of the destination device [R2.C2.z] and include the calculation results in the frame header—that is, D1 = [(R2−R1),(C2−C1).z].
b. The source device or router selects the neighboring router with the closest distance to the destination device and transmits the data frame to the selected router. For example, the distance between the current location and destination device is D1 = [(Rd1 = (R2−R1)).Cd1 = (C2−C1).z], and the distance between the neighboring router and the destination device is Dn(Rdn.Cdn.0). The source device or router selects the neighboring router with the address [(|Rdn|<=|Rd1|).(|Cdn|<=|Cd1|.n] and transmits the data frame to the selected router.
c. If there are two neighboring routers satisfying the condition [(|Rdn|<=|Rd1|).(|Cdn|<=|Cd1|).n], the source device or router randomly selects the next router and transmits the data frame to the selected router.
d. Whenever the data frame moves one hop to the next router, the router decreases the row ID or column ID of D1 by one, based on the direction of movement.
e. Steps b–d are repeated until Rd1 = 0 and Cd1 = 0.

The benefits of the TSMR protocol can be summarized as follows: (i) because a multi-path route between the source device and the destination device exists, the link failure is not influenced in the entire network; (ii) because the control message for the routing protocol is unnecessary, the TSMR protocol can reduce its energy consumption and control overhead; and (iii) using Steps a and c, the traffic load in the network is distributed.

### 3.5. Handoff

To maintain connection with the network, the leaf device periodically checks the link quality with the parent router through the received beacon frame. If the RSSI of the current parent router is lower than the defined threshold (equivalent to −70 dB), the leaf device searches for another router with a higher RSSI. When a router with a higher RSSI exists, the device can participate in the new network by sending an association-request frame to the new router.

**Figure 11:**
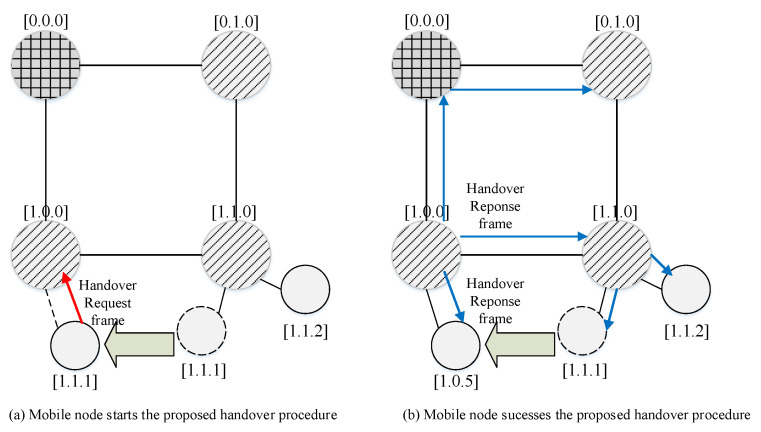
Example of the proposed handover procedure.

Figure 11 shows an example of the proposed handover procedure.

**Figure 12:**
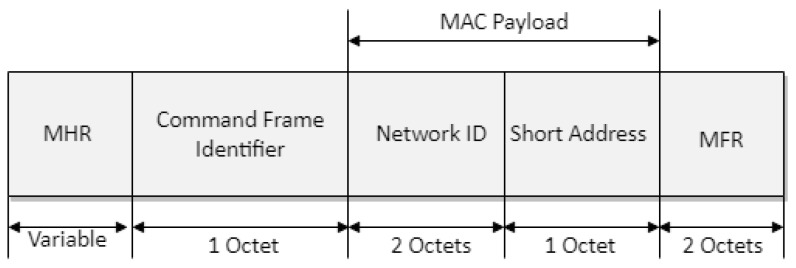
The format of the proposed handover-request frame.

In Figure 11a, a mobile node [1.1.1] moves into the communication area of the router [1.0.0] and starts the handover process by sending a handover-request frame to the router [1.1.0]. Figure 12 shows the format of the proposed handover-request frame.

**Figure 13:**
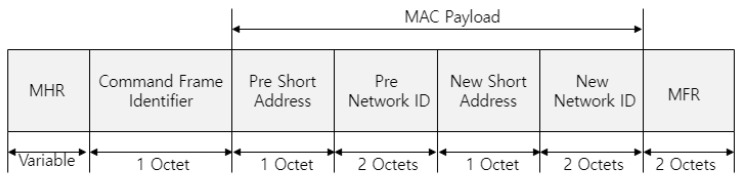
The proposed handover-response frame structure.

The router [1.1.0] that receives the handover-request frame allocates a new address [1.0.1] to the mobile node and broadcasts the handover-response frame to all neighboring nodes. Figure 13 shows the proposed handover-response frame structure.

The mobile node that receives the handover-response frame starts the data transmission process. The base station that receives the handover-response frame relays the received frame to the neighboring router [0.1.0]. 

In the TSMR protocol, because coordinators rebroadcast the received handover-response frame to share information regarding the movement of a mobile device, some intermediate routers can recognize the new address of the moved device. Consequently, the source device can transmit a data frame to the new destination through an intermediate router that obtains the new address of the moved destination device, although it does not recognize the handover of the destination device. Also, if there is no response from the leaf device, the router buffers the packet. Upon receiving the handover response frame, the buffered packets are forwarded to the router that sent the handover response frame, and the router transmits the packets that are forwarded to the newly subscribed leaf device. This process cannot completely prevent packet loss, but it can minimize loss.

When the router moves, it broadcasts a handover-notification frame to its leaf device. The leaf devices that receive it transmits dummy data to the router, and the router selects the leaf device with the highest RSSI to the new router. The selected device changes its leaf ID to zero, and the legacy router performs a handover process.

## 4. Performance Evaluation

We used the OMNeT++ simulator [73] to evaluate the performance of the TSMR. The common simulation parameters are summarized in Table 1, and we adopted the energy model of CC2630 [74]. The simulation model of the IEEE 802.15.4 in [75] is good since it provides several MAC layer service primitives, channel scan operations, beacon management functions, and WPAN management functions. Therefore, it has been used in several studies to evaluate the performance of the TSMR. In [75], it consisted of the following modules—that is, an application layer implementing the traffic generator, battery module, network module, and physical layer module. The environmental parameter settings were determined by adjusting the variables in the omnetpp.ini configuration file of the model. The simulations were conducted in beacon-enabled mode, and all packets required an ACK frame. The devices were placed randomly on a 1000 × 1000 m plane. The simulations were run for 1000 s, with the number of devices in the network being 400. For accuracy, the same configuration simulation was performed 20 times.

In this simulation, we compared the TSMR with the IEEE 802.15.4 [1], RPL [54], and MeshMAC [61] schemes. For mesh networking using the IEEE 802.15.4 standard, we applied the AODV routing protocol in the IEEE 802.15.4 non-beacon enable mode.

**Figure 14:**
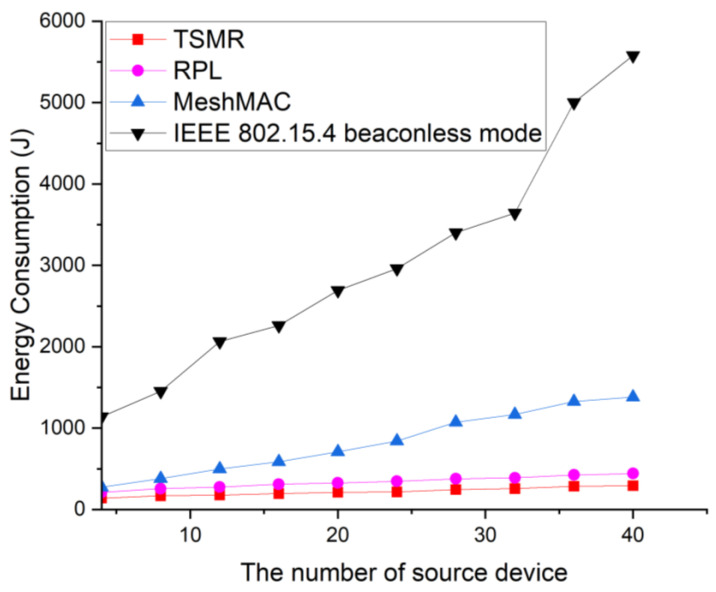
Energy consumption of the network as a function of the number of source devices.

Figure 14 shows the energy consumption as a function of the number of source devices.

In this simulation, the transmission pair was randomly selected. For the IEEE 802.15.4 standard, devices participating in routing consume significant energy to establish and maintain routes. Therefore, the IEEE 802.15.4 standard provides the lowest energy performance. MeshMAC provides beacon scheduling techniques to reduce power consumption, and all devices operate in duty cycles. Therefore, the MeshMAC provides better energy performance than the IEEE 802.15.4 standard. However, since intermediate devices on the path simply forward data frames to the next hop device without a fixed path, even unnecessary devices participate in routing. Thus, many intermediate devices consume energy to relay the data frame to the destination. RPL transmits packets by bypassing the route, generating many control packets for flooding-based path discovery and topology maintenance, which can cause network congestion. Therefore, the RPL consumes more power than the TSMR. TSMR provides the best energy performance because it does not generate control packets for routing frames, and control packets for path maintenance do not occur except for packets for handover.

**Figure 15:**
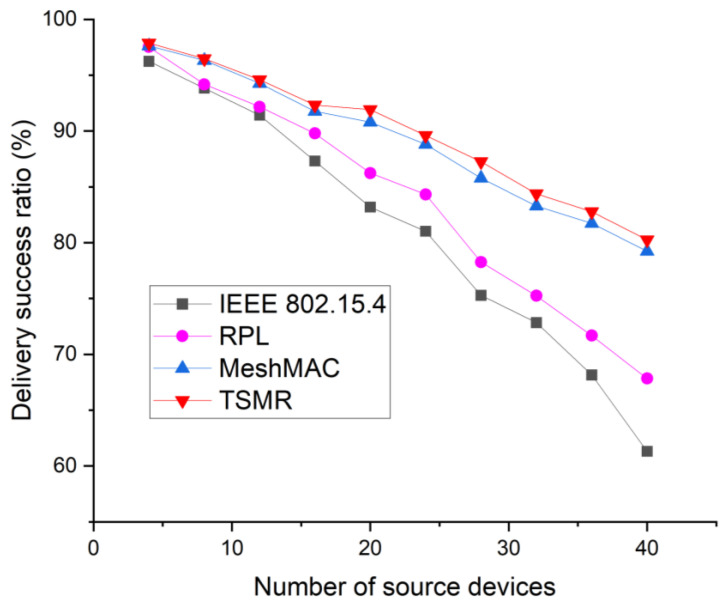
Delivery success ratio as a function of the number of source devices.

Figure 15 shows the delivery success ratio as a function of the number of source devices. The delivery success ratio represents the ratio of the number of packets successfully arriving at the destination among data packets generated by the source node. Thus, this performance indicator implies the reliability of the data packet. As the number of source devices increases, many devices generate a large amount of control packets to set and maintain routing paths. In addition, because they participate in routing protocols and transmit data packets, the delivery success ratio of the IEEE 802.15.4 standard decreases and the collision probability increases significantly. Because packets in the RPL tend to bypass the route, they are concentrated on nodes close to the route, resulting in transmission failures around the route. Especially for IEEE 802.15.4 and RPL, the gap is much wider than the mesh topology. This is because it transmits control packets in a flood manner for route discovery and generates many control packets to maintain the set path, resulting in network congestion and packet drop. However, since MeshMAC and TSMR do not generate control packets for routing and do not transmit control packets for routing, the probability of collision between the two protocols is lower than that of the other protocols. As a result, the delivery ratio of TSMR and MeshMAC is superior to that of the IEEE 802.15.4 standard and the RPL.

**Figure 16:**
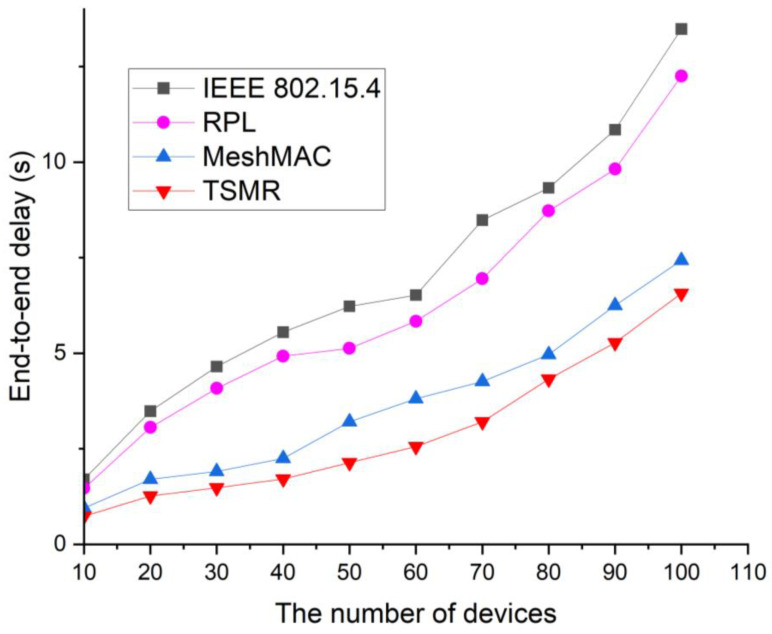
End-to-end delay as a function of the network size.

Figure 16 shows the end-to-end delay as a function of network size.

In this simulation, all leaf devices in the network transmit data every second. As the number of devices in the network increases, traffic in the network increases. In the IEEE 802.15.4 standard, devices on the routing path transmit many control packets to set and maintain paths, and since all devices on the network participate in competition for data transmission, devices on the routing path have limited opportunities for data transmission and increase the probability of data collision. The end-to-end delay in the RPL is clearly longer than the delay in the mesh topology because it causes a bypass problem in which packets are passed to the root along a predefined path until they reach a common ancestor or root.

**Figure 17:**
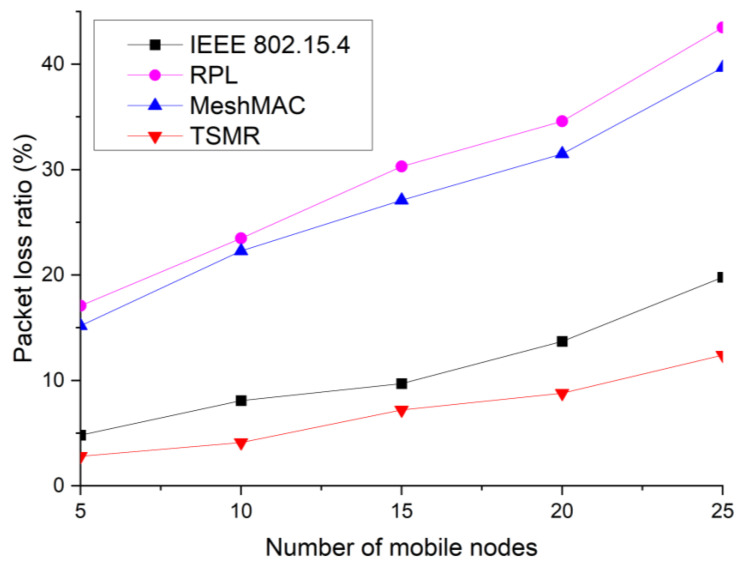
Packet loss ratio as a function of the number of mobile nodes.

Figure 17 illustrates the packet loss rate when the number of mobile leaf nodes is varied.

In this simulation, the mobile leaf device moves at a speed of 1 m/s. In Figure 17, the MeshMAC and RPL methods exhibit the worst performance because they do not consider the mobility of router devices. In the IEEE 802.15.4 standard, the device performs a path recovery process in the AODV protocol to reduce packet loss. TSMR experiences some packet loss during the handover process, but the impact of device mobility is minimal.

**Figure 18:**
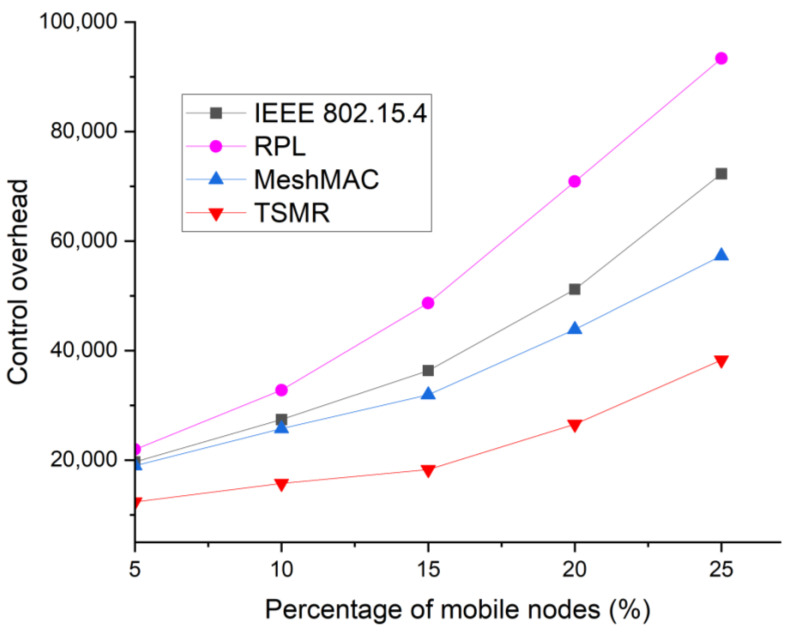
Control overhead as a function of the number of mobile nodes.

Figure 18 shows the control overhead according to the number of devices moving. In Figure 18, the control overhead represents the total number of packets used for route discovery. This overhead shows the degree of energy consumption of the protocol because control packets cause energy consumption of the device and shorten the network life. When the device moves, a large number of devices must send control packets and reset the path to find the moving device. Thus, as the number of mobile devices increases, more control overhead occurs. TSMR also shows that as the number of devices moving increases, the control overhead increases, but the control overhead decreases because the number of control packets for resetting the routing path can be reduced.

## 5. Conclusions

In this paper, we proposed an IEEE 802.15.4 MAC layer-based cross-layer protocol for mesh routing. The TSMR can provide robust connectivity and efficient route discovery procedures from the base station to mobile and static leaves, and vice versa. Additionally, the proposed scheme provides fast and efficient association/reassociation procedures for the mobile nodes while limiting the impact of the signaling overhead to support the device’s mobility. Finally, the TSMR can provide an efficient and distributed address-allocation algorithm and improve the network reliability. We evaluated the performance of the IEEE 802.15.4 standard, RPL, MeshMAC, and TSMR through simulation using realistic radio models. Simulation results indicate that the TSMR can improve reliability and reduce end-to-end delays. The proposed protocol could be directly applied with a small overhead to the current IEEE 802.15.4 systems.

## Figures and Tables

**Table 1:** Simulation parameters.

Parameters	Value
Transmission power (dBm)	3.5
Bandwidth	2.8 MHz
Synchronization mode	Beacon-enabled
Carrier sense sensitivity	−85 dBm
Channel number	11
IEEE 802.15.4 Header Length	22 bytes
Packet Size	50 bytes
RX current consumption	5.9 mA
TX current consumption	9.1 mA
IDLE current consumption	0.550 mA
Sleep current consumption	0.001 mA
BO	10
SO	4

## Data Availability

Not applicable.
